# Case Series of Multisystem Inflammatory Syndrome in Neonates (MIS-N) With a Link to Coagulopathy

**DOI:** 10.7759/cureus.49681

**Published:** 2023-11-29

**Authors:** Deepthi Siramdas, Peddi Anudeep, Preethi Subramanian, Sudharshanraj Chitgupikar

**Affiliations:** 1 Pediatrics, MediCiti Institute of Medical Sciences, Hyderabad, IND

**Keywords:** coagulopathy, covid-19, intracerebral bleeds, intravenous immunoglobulin, steroids

## Abstract

Introduction

Multisystem inflammatory syndrome in neonates (MIS-N) is an emerging clinical entity observed in neonates born to mothers with exposure to the SARS-CoV-2 virus before or during the antenatal period.

Methods

We report 18 neonates diagnosed with MIS-N from retrospectively collected data. A total of 18 neonates (13 term and five late-preterm; 10 males) admitted to the neonatal intensive care unit (NICU) of a tertiary care medical institute, between June 2021 to November 2022, were diagnosed with MIS-N.

Results

The median age of presentation of the 18 neonates was 1.5 days of life. All the neonates were positive for SARS-CoV-2 IgG antibodies and had elevated D-dimer levels. Respiratory system involvement was the most common (12 of 18 neonates: 66.67%). Ten out of 18 neonates (55.55%) had coagulopathy. Seven of the ten neonates with coagulopathy had central nervous system (CNS) involvement as seizures and/or intracerebral infarcts/bleeds. Cardiovascular and gastrointestinal system involvement was observed in nine (50%) and seven (38.89%) neonates, respectively. One out of 18 neonates died due to intraventricular and cerebral hemorrhage. The mortality rate was 5.55% (n=1). Ten of 18 neonates with coagulopathy required fresh frozen plasma along with repeated therapeutic doses of injection vitamin K. Eight neonates (44.44%) required human intravenous immunoglobulin (IVIG), and three neonates (16.67%) required steroids and IVIG for recovery along with supportive care.

Conclusion

Coagulopathy can be one of the salient features of presentation in MIS-N. In the immediate post-pandemic era, it is essential that MIS-N is considered in the differential diagnosis of neonates presenting with intracerebral bleeds/infarcts. IVIG and steroids might play an important role in the treatment of neonates with MIS-N.

## Introduction

Multisystem inflammatory syndrome in neonates (MIS-N) is a recently observed clinical entity in neonates born to mothers who have had exposure to the SARS-CoV-2 virus during their antenatal period [1-2]. Multiple case reports on MIS-N from different parts of the world have been published in 2021, but it is to be noted that the pathophysiology of MIS-N is not completely understood [3]. This disease is hypothesized to be the result of certain antibodies formed in the mother as a defense against SARS-CoV-2 that crosses the placenta and acts against the antigens of the fetus/neonate. The clinical presentation, laboratory features, and diagnostic criteria for MIS-N are still evolving and need further research.

More et al. in 2022 provided a diagnostic criterion for MIS-N which is as follows: i) onset of symptoms anytime from birth to ≤28 days of life [2]. ii) fever along with the involvement of ≥2 systems (i.e., cardiac, renal, respiratory, hematologic, gastrointestinal, dermatologic, and neurological) requiring hospitalization (fever was not taken as a mandatory symptom as neonates do not commonly present with fever). iii) evidence of raised inflammatory markers including any one of the following: c-reactive protein (CRP), procalcitonin (PCT), ferritin, D-dimer, IL-6, and pro-BNP (brain natriuretic peptide). iv) evidence of SARS-CoV-2 antibodies (SARS-CoV-2 IgG) in the mother and neonate. v) exclusion of alternate diagnoses with similar clinical presentations, like perinatal asphyxia, sepsis confirmed by blood culture, and maternal lupus resulting in atrioventricular conduction abnormalities in the fetus and neonate.

We report a case series of eighteen neonates diagnosed with MIS-N in our institute and their clinical and biochemical profile, management, and outcome.

## Materials and methods

A total of 18 neonates (13 term and five late-preterm; 10 males and eight females) admitted to the neonatal intensive care unit (NICU) of a tertiary care medical institute, between June 2021 and November 2022, were diagnosed with MIS-N. The demographic characteristics and clinical profile of the neonates are provided in Table 1. Various researchers have attempted to come up with diagnostic criteria for MIS-N based on the multisystem inflammatory syndrome in neonates (MIS-C) criteria [1-2]. Based on the criteria mentioned by More et al., the neonates were diagnosed with MIS-N. After diagnosis of MIS-N, the neonates were classified into the following categories: most likely MIS-N, when all criteria mentioned above are present, and possible MIS-N, when there is an unusual clinical presentation, suspicious of MIS-N, not meeting all specified diagnostic criteria but there is no other explainable cause [2].

**Table 1:** Demographic characteristics, clinical profile, and investigations of the neonates *Lab controls were APTT, 30-40 sec; PT, 11-15 sec. SARS-CoV-2, severe acute respiratory syndrome coronavirus; DOL, day of Life; CPAP, continuous positive airway pressure; IVIG, intravenous immunoglobulin; MIS-N, multisystem inflammatory syndrome in neonates; CBP, complete blood picture; CRP, c-reactive protein; CSF, cerebrospinal fluid analysis; GCMS, gas chromatography mass spectrometry; TMS, tandem mass spectrometry; NG, nasogastric; SGOT, serum glutamate oxaloacetate transferase; SGPT, serum glutamate pyruvate transferase; ABG, arterial blood gas; EEG, electroencephalogram; NSG, neurosonogram; 2DECHO, 2D echocardiogram; APLA, antiphospholipid antibodies; FDP, fibrin degradation product; pro-BNP, brain natriuretic peptide; WBC, white blood cells; APTT, activated partial thromboplastin time

Neonate case no.	Age at presentation/ sex/birth weight (g)/gestational age	Maternal SARS-CoV-2 (spike antigen) IgG status/RT PCR for COVID-19/vaccination status	Neonatal SARS-CoV-2 (spike antigen) IgG status	Salient manifestations	Investigations	Diagnosis/outcome
1.	1st DOL/F/3200 g/39+6 week	Positive/negative/unvaccinated	Positive	Seizures on 1st DOL; respiratory distress on 2nd DOL; hypotension & shock: 2nd DOL	CBP: Hb: 12.7 g/dL; WBC & platelets: normal; CRP: normal (<6 mg/L); blood C/S: no bacterial growth. APTT*-45.9 sec; PT*: 15.8 sec; INR: 1.17; D-dimer: 1427 ng/mL (normal value <500 ng/mL); pro-BNP: 7042 pg/mL (10-115 pg/mL); APLA: normal; MRI - acute infarct in left parietal region, subdural hemorrhage in parieto occipital region & subarachnoidal hemorrhage in posterior parietal region; 2D Echo: normal; chest x-ray: normal	Most likely MIS-N/discharged
2.	1st DOL/M/3115 g/ 39+1 week	Positive/negative/one dose	Positive	Vesicles and bullae on skin not involving palms and soles on 1st DOL; blood in NG aspirates on 1st DOL	CBP: Hb: 16.6 g/dL; WBC & platelets: normal; CRP: normal; skin-fluid culture and sensitivity: no microbial growth; blood C/S: no bacterial growth. TORCH (IgM profile): negative; Tzanck smear: negative; APTT-50.9 sec; PT: 15.8 sec; INR: 1.2; D-dimer: 2324 ng/mL; fibrinogen & FDP: normal; 2D Echo, NSG & MRI brain: normal; chest x-ray: normal	Most likely MIS-N/discharged
3.	2nd DOL/F/2750 g/38+3 weeks	Positive/negative/one dose	Positive	Persistent respiratory distress requiring CPAP for 5 days; bloody NG aspirates from 1st DOL; feed intolerance 48 hours of life	CBP: Hb: 16.3 g/dL; WBC & platelets: normal; CRP: 68 mg/L (normal: <6 mg/L); blood C/S: no bacterial growth. Chest x-ray: normal; APTT-41.8 sec; PT: 17.3 sec; INR: 1.3; D-dimer: 1495 ng/mL; 2D Echo & NSG: normal	Most likely MIS-N/discharged
4.	1st DOL/M/2910 g/39+5 weeks	Positive/negative/one dose	Positive	Persisting respiratory distress for >72 hours; rash in b/l Inguinal areas on Day 1	CBP: Hb: 17.9 g/dL; WBC & platelets: normal; CRP: normal; blood C/S: no bacterial growth; APTT-40.5 sec; PT: 15.9 sec; INR: 1.1; D-dimer: 1072 ng/mL; SGPT: 95 IU/L; SGOT: 135 U/L (elevated); 2D Echo & NSG: normal. Chest x-ray: normal	Most likely MIS-N/discharged
5.	1st DOL/M/1710 34+3 week	Positive/negative/unvaccinated	Positive	Respiratory distress persisting for >72 hours of life since birth but not requiring surfactant; feed intolerance-altered aspirates from 2nd to 6th DOL	CBP: Hb: 15.4 g/dL; WBC & platelets: normal; CRP: normal; blood C/S: no bacterial growth; APTT - 40.5 sec; PT: 14.3 sec; INR: 0.8; FDP: normal; D-dimer: 1383 ng/mL; NSG: normal. Chest x-ray: no infiltrates/ opacities; well inflated; blood C/S: no bacterial growth	Most likely MIS-N/discharged
6.	1st DOL/M/2345 g/37+2 weeks	Positive/negative/one dose	Positive	feed intolerance-abdominal distension; bloody altered aspirates from 1st DOL to 4th DOL	CBP: Hb: 22.5 g/dL; WBC & platelets: normal; CRP: normal; blood C/S: No bacterial growth; APTT - 48.5 sec; PT: 19.2 sec; INR: 1.3; FDP: normal; D-dimer: 1383 ng/mL; NSG: normal; chest x-ray and abdomen: normal lung fields with normal gas shadows of intestines	Most likely MIS-N/discharged
7.	2nd DOL/M/2325 g/39+1 weeks	Positive/negative/one dose	Positive	Respiratory distress from 2nd DOL; fever from 3rd-6th DOL; irritable and dull sensorium from 2nd DOL	CBP: Hb: 18.1 g/dL; WBC & platelets: normal; CRP: normal; blood C/S: no bacterial growth; APTT - 41.5 sec; PT: 14.2 sec; INR: 1.1; FDP: normal; D-dimer: 4023 ng/mL; ferritin: 467 ng/mL (16-245 ng/mL); NSG: normal. Chest x-ray: no specific abnormalities	Most likely MIS-N/discharged
8.	1st DL/F/1550 g/36 weeks	Positive/negative/unvaccinated	Positive	Respiratory distress and blood in NG aspirates; shock from Day 1 of life	CBP: Hb: 19.1 g/dL; WBC & platelets: normal; CRP: normal; blood C/S: no bacterial growth; APTT - 40.5 sec; PT: 26.2 sec; INR: 1.85; FDP: normal; D-dimer: 865 ng/mL; NSG: normal; chest x-ray: normal	Most likely MIS-N/discharged
9.	1st DOL/F/2950 g/39 weeks	Positive/negative/unvaccinated	Positive	5 episodes of vomiting on 1st DOL; abdominal distension from 1st to 6th DOL; blood in NG aspirates from 2nd to 4th DOL; 3 episodes of seizures on 6th DOL	CBP: Hb: 16.7 g/dL; WBC & platelets: normal; CRP: normal; blood C/S: no bacterial growth; APTT - 32.5 sec; PT: 16.3 sec; INR: 1.12; D-dimer: 1023 ng/mL; stool for occult blood: positive; NSG & EEG: normal; chest x-ray: normal; x-ray abdomen erect view: distended bowel loops with no signs of obstruction	Most likely MIS-N/discharged
10.	1st DOL/F/3040 g/37+3 weeks	Positive/negative/unvaccinated	Positive	Respiratory distress at birth; feed intolerance on day 4; ecchymosis of face; hematuria, bleeding from multiple sites on 5th DOL; seizures on day 10	CBP: Hb: 13.7 g/dL; Repeat Hb on 3rd DOL: 11 g/dL; WBC & platelets: normal; CRP: 96 mg/L (Normal: <6 mg/L); blood C/S: no bacterial growth; APTT - 56.5 sec; PT: 19.3 sec; INR: 1.4; D-dimer: 7023 ng/mL; FDP: elevated; fibrinogen: elevated; stool for occult blood: positive; NSG–IVH on day 4 of life; MRI on day 8 - intra parenchymal hemorrhages & hemorrhagic infarcts in various stages (Figure 1), large subdural hematoma along frontoparietal, kinking of optic nerves & flattening of sclera	Most likely MIS-N (Died)
11.	2nd DOL/M/3100 g/38+1 weeks	Positive/negative/unvaccinated	Positive	Respiratory distress from 2nd DOL for 3 days; focal clonic seizures involving Rt upper and lower limbs (5 episodes)	CBP: Hb:15.3 g/dL; WBC & platelets - normal; CRP: 5 mg/L; blood C/S: no bacterial growth. Chest x-ray: normal. CSF: cell count - 3/mm^3^, glucose-51 mg/dL, and protein-69 mg/dL. CSF C/S: no microbial growth. ABG: normal; mention values of pCO_2_ and HCO_3_ 2D Echo, EEG & GCMS (urine) & TMS (blood): normal; MRI showed acute brain infarct in the left frontal lobe extending into the parietal region, anterior limb, and genu of internal capsule with surrounding edema, suggestive of arterial ischemic stroke (Figure 2); APLA levels in mother: normal give values; serum homocysteine: 7.72 μmol/L (normal). Serum protein C&S at 3 months of life: normal	Perinatal arterial ischemic stroke/possible MIS-N/discharged
12.	1st DOL/M/2850 g/37+3 weeks	Not done/negative/unvaccinated	Positive	Respiratory distress persisting >72 hours since birth; fever on 2nd DOL; blood in nasogastric aspirates from day 2; shock	CBP: Hb: 18.3 g/dL; WBC: normal; platelets: 1,30000/cu.mm; repeat CBP on 3rd DOL: Hb: 13.2 g/dL; platelets: 142000/ cu.mm; WBC: normal; CRP: normal; APTT - 51.8 sec; PT: 20.9 sec; INR: 1.3; D-dimer: 778 ng/mL; 2DEcho: normal study; CT: subdural hemorrhage; chest x-ray: normal	Possible MIS-N/discharged
13.	1st DOL/M/2150 g/36 weeks	Not done/negative/one dose	Positive	Respiratory distress from birth to 4th DOL (not requiring surfactant); blood in NG aspirates on 1st DOL; hypotension on 2nd DOL; malena on 2nd DOL	CBP: Hb: 17.0 g/dL; WBC & platelets: normal; CRP: normal; APTT-48.3 sec; PT: 17.9 sec; INR: 1.4; stool for occult blood: positive; D-dimer: 845 ng/mL; SGOT: 91 U/L; SGPT: 123 U/L; 2DEcho & NSG: normal. Chest x-ray: lung fields normal	Possible MIS-N/discharged
14.	1st DOL/M/2880 g/39+6 weeks	Not Done/negative/one dose	Positive	Blood in NG tube aspirates on 1st DOL; multiple episodes of seizures on 1st DOL; impaired circulation on 2nd DOL	CBP: Hb: 16.8 g/dL, WBC & platelets: normal; CRP on 1st DOL: within normal limits; D-dimer: 943 ng/mL; APTT - 52.6 sec; PT: 18.9 sec; INR: 1.4; APLA levels in mother: normal; EEG-left temporal sharp & slow wave discharges; MRI brain-acute infarct in post-parietal region, acute lacunar infarct in left frontal region; 2DEcho: normal	Possible MIS-N/discharged
15.	1st DOL/F/1400 g/34+6 weeks.	Not done/negative/one dose	Positive	Resp. distress from 1st to 4th DOL; shock on 2nd DOL; seizures on day 3	CBP: Hb: 15.3 g/dL; WBC & platelets: normal; CRP: normal; APTT - 52.1 sec (30-40 sec: normal); PT: 21.1 sec (11-15 sec: normal) INR: 1.4; serum calcium and electrolytes: normal; D-dimer: 1723 ng/mL; pro-BNP: 1540 pg/mL; FDP and fibrinogen: normal; CSF analysis & NSG: normal. Chest x-ray: normal	Possible MIS-N/discharged
16.	1st DOL/M/2065 g/34+4 weeks	Not done/negative/one dose	Positive	Feed intolerance on 1st DOL; hypotension on 4th DOL	CBP: Hb: 19.0 g/dL; WBC & platelets: normal; CRP: normal; APTT-44.5 sec; PT: 16.9 sec; INR: 1.1; D-dimer: 1657 ng/mL; FDP and fibrinogen: normal; NSG: normal. Chest x-ray: normal; blood C/S: no bacterial growth	Possible MIS-N/discharged
17.	1st DOL/F/2115 g/39+3 weeks	Not done/negative/one dose	Positive	Multiple petechiae all over the body including face at birth; altered aspirates from NG tube with vomiting from 12 hours of life	CBP: Hb: 14.0 g/dL; WBC: 18000/cu.mm & platelets: 1.67 Lakhs/cu.mm; CRP: normal; APTT - 43.6 sec; PT: 19.9 sec; INR: 1.2; D-dimer: 1330 ng/mL; fibrinogen & TORCH (IgM profile): negative; NSG: normal; blood C/S: no bacterial growth	Possible MIS-N/discharged
18.	1st DOL/F/2925 g/38+2 weeks	Not done/negative/fully vaccinated	Positive	Respiratory distress persisting beyond 72 hours; feed intolerance: from 2nd to 4th DOL	CBP: Hb: 14.0 g/dL; WBC & platelets: normal; CRP: normal; blood C/S: no bacterial growth; APTT - 40.6 sec; PT: 13.9 sec; INR: 1.07; D-dimer: 1572 ng/mL; NSG: normal; chest x-ray: normal	Possible MIS-N/discharged

**Figure 1:**
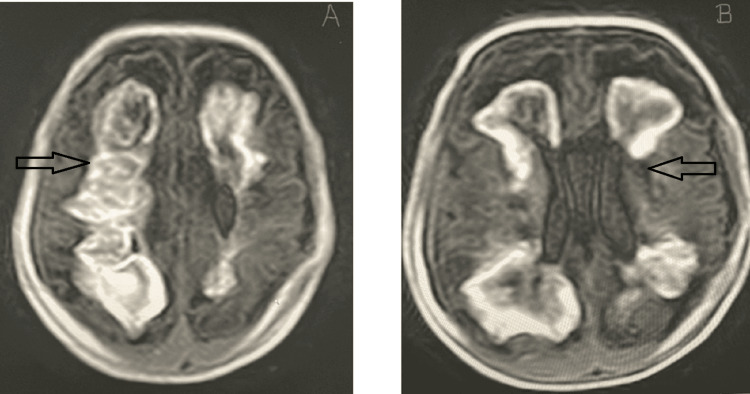
(a) T1-weighted images depicting heterogenous confluent areas of hemorrhages, and (b) hemorrhage involving periventricular area and corona radiata in case 10

**Figure 2:**
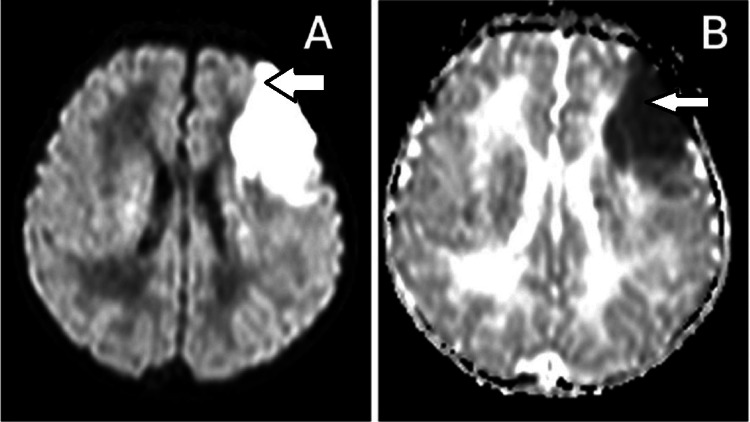
(a) Diffuse restriction on diffusion-weighted imaging, and (b) reversal on apparent diffusion coefficient depicting acute brain infarct in the left frontal lobe extending into the parietal region, anterior limb, and genu of the internal capsule with surrounding edema in case 11

## Results

The detailed clinical presentation and investigations are shown in Table 1. Investigations were carried out according to the presenting symptoms. Common clinical conditions like early onset sepsis and necrotizing enterocolitis were ruled out by relevant investigations like CRP, blood for culture and sensitivity, stool for occult blood, and chest and abdominal x-rays.

When the clinical scenario suggested multisystem involvement, quantitative immunoassay for SARS-CoV-2 IgG antibodies, to identify neutralizing antibodies against S1/S2 spike antigens of the virus, was done for all the neonates and 11/18 mothers. The antibodies were positive in all neonates and 11 mothers. Four mothers refused consent for the test and the rest three denied it due to affordability issues. The D-dimer assay done for all the neonates showed raised titers in all neonates [2,4]. The other inflammatory markers like fibrin degradation product (FDP), fibrinogen, ferritin, and pro-BNP were done as per affordability. 

Most of the neonates (14/18; 77.78%) presented with bleeding manifestations such as blood in nasogastric aspirates, melaena, intracranial bleeds (intracerebral, periventricular, subarachnoid, subdural bleed), and rash in the skin but with normal platelet count. The absence of laboratory parameters to support sepsis led us to evaluate coagulopathy with a coagulation profile. Among the 18 neonates, 10 neonates (55.56%, cases 2, 3, 6, 8, 10, 12 to 15, 17) had altered coagulation profiles with either prolonged prothrombin time (PT) or APTT (activated partial thromboplastin time) or both [5]. Liver function test and renal function test were done as per clinical need. Trans-fontanelle ultrasonogram (USG or neurosonogram) was done for all neonates, while CT/MRI brain was done, where indicated (clinical presentation of seizures/encephalopathy/abnormal tone or when neurosonogram was abnormal). Seven neonates had central nervous system (CNS) involvement in the form of seizures due to intracerebral infarcts/bleeds (cases- 1, 9, 10, 11, 12, 14, and 15). Neonates with cerebral infarcts were evaluated to rule out prothrombotic states like antiphospholipid antibodies (APLA), protein C, and protein S.

The neonates were provided with partial parenteral nutrition and supportive medications such as oxygen by nasal prongs (n=8; 44.44%) and/or continuous positive airway pressure (CPAP) (n=6; 33%). Six neonates (33%) received antiepileptics, namely, phenobarbitone and/or phenytoin/levetiracetam for seizures. All the 14 neonates with bleeding manifestations received therapeutic doses of vitamin K injection. Ten of these were transfused with fresh frozen plasma as they had major bleeds. Seven neonates (38.89%) with clinical features of delayed capillary refill time and/or hypotension received inotropes such as dobutamine and/or dopamine infusion at 5-10 mg/kg/min. One neonate (case 10) required ventilator support due to recurrent seizures, refractory to multiple antiepileptics (phenobarbitone, phenytoin, levetiracetam, and infusion of midazolam). This neonate had multiple intracerebral (parenchymal and ventricular) bleeds, developed disseminated intravascular coagulation (DIC) and succumbed to the illness on the 10th day of life. All other neonates survived and were discharged home.

Eight neonates (44.44%) (cases 1, 2, 3, 7, 9, 11, 13, 15) in this series received human intravenous immunoglobulin (IVIG) - 2 g/kg over 48 hours as slow infusion. Three of these neonates (16.67%) (cases 1, 9, and 13) received intravenous dexamethasone 0.5 mg/kg/day in two divided doses for three days in addition to IVIG. The mean age at which IVIG was administered was 4.2±0.8 days. There were no adverse drug reactions to IVIG in any of these neonates. All eight neonates who received IVIG showed clinical improvement and became stable by the end of 48-72 hours. The decision to treat neonates with IVIG and/or steroids was taken by the treating pediatrician on the grounds of inadequate or less-than-expected clinical improvement with supportive treatment alone.

## Discussion

MIS-C was observed in children from across the globe as the first wave of COVID-19 infection started to decrease. The initial report was from the United Kingdom in April 2020 [6]. A similar pattern of multisystem involvement with raised inflammatory markers in neonates was first reported in Qatar in November 2020 [7]. The diagnosis of MIS-N was sought once the symptoms that the neonates presented with did not fit the common differential diagnosis. Clinical suspicion plays a strong role in the diagnosis of MIS-N as there are no set patterns or specific symptoms. The most common systems in the present case series included respiratory (12/18; 66.67%), hematological (10/18; 55.56%), cardiovascular system (CVS) (9/18; 50%), followed by gastrointestinal (7/18; 38.89%) and CNS (7/18; 38.89%). This pattern of cardiorespiratory system involvement is commonly observed in other studies too [1-2,8]. In the present series, hematological system involvement is high, which could also explain the higher number of neonates (38.89%; n=7) presenting with CNS involvement (intracerebral bleeds/infarcts). When the neonates presented with intracerebral infarcts/bleeds, most of the common differential diagnoses like sepsis, DIC, APLA-induced vasculitis, and TORCH infections were ruled out, and evaluation for coagulopathy as a component of MIS-N presentation was sought. To the best of our knowledge, this case series has the highest number of neonates with coagulopathy (55.56%) and CNS involvement (38.89%) compared to the previously published literature. A comparison of published case series on MIS-N from India and abroad is provided in Table 2. As we searched the literature, (including literature search done till March 31, 2023), there were multiple case series and case reports reported since 2020. Among the neonates (n=98) reported in the literature (Table 2), most were preterm neonates (54.1%, n=54 out of 98 in Table 2); the majority of neonates had early MIS-N, that is, within the first 72 hours of life. The most common organ system involved was the respiratory and CVS in the literature (61.22%; n=60 and 55.1%; n=54, respectively). This was akin to the present case series where 12 out of the 18 neonates had respiratory symptoms. In the literature, 18 neonates (18.37%) had CNS involvement in the form of encephalopathy and seizures while the present series has 38.89% CNS involvement (n=7). Coagulopathy was observed in 15 neonates (15.31%) in the literature, while 55.56% (n=10/18) in the present case series had coagulopathy (altered coagulation profile). Fever was observed only in 42 (42.8%) neonates in the literature. There are at present no standard guidelines for the treatment of neonates with MIS-N. The experiences and treatment regimens applied to MIS-C patients have been used and extrapolated for neonates [7-10]. Apart from the supportive treatment, IVIG and steroids were used in 67 (68.36%) and 75 (75.31%) neonates, respectively, in the literature. The majority of neonates had favorable outcomes and were discharged home and only 7 (7.14%) neonates died among the 98 in the literature. In the present case series, eight out of 18 neonates (44.44%) received IVIG. In our institute, IVIG was the initial choice of treatment, when supportive treatment alone was not helpful.

**Table 2:** Comparison of case series of MIS-N from India and abroad DOL, day of life; IVIG, intravenous immunoglobulin; LMWH, low molecular weight heparin; CNS, central nervous system; r-TPA, recombinant tissue plasminogen activators; GI, gastrointestinal; MP, methyl prednisolone; CVS, cardiovascular system; MIS-N, multisystem inflammatory syndrome in neonates

S. No	Author/place of study	Study period	Number of neonates (n)	Median age at presentation	Most common organ system involved	Treatment provided	Outcome (mortality)
1.	Pawar et al., kohlapur, India [1]	September 1, 2020-April 30, 2021	20	2nd DOL	Cardiac (90%)	IVIG & steroids (100%); LMWH - 70%	2 (10%)
2.	More et al., Western India [2]	October 2020-March 2021	20	3.5 DOL	Respiratory (55%)	IVIG: 8 (40%); steroids: 17 (85%)	2 (10%)
3.	Amonkar et al., Mumbai [11]	2020	1	3rd DOL	CNS skin (ischemia of one limb)	Steroids, r-TPA, surgical embolectomy	Neonate survived but underwent amputation of one leg
4.	Magboul et al., Qatar [7]	2020	1	14th DOL	Liver, coagulopathy	IVIG, steroids, anakinra	Favorable
5.	Balleda et al., Andhra Pradesh, India [9]	June 1-September 30, 2021	18	Not available	Respiratory (83.30%)	IVIG & steroid: 18 (100%) LMWH: 9 (50%)	1 (5.55%)
6.	Kappanayil et al., Kochi, India [12]	2021	1	24th DOL	Cardiovascular and skin	IVIG steroids heparin	Favorable
7.	Diggikar et al., Bengaluru [13]	2021	1	7th DOL	GI CNS	Steroids (dexa & MP) IVIG enoxaparin	Favorable
8.	Diwakar et al., Jharkhand [14]	2021	1	18th DOL	Fever, skin, GI	IVIG, aspirin	Favorable
9.	Agarwal et al., Haryana [15]	2021	1	2nd DOL	Fever, GI, cardiac & skin	IVIG steroids enoxaparin aspirin	Favorable
10.	Borkotoky et al., Assam [16]	2021	1	7th DOL	Respiratory, skin, GI	Steroids, sildenafil, furosemide	Favorable
11.	Saha et al., Kolkata [17]	2021	1	8th DOL	Fever, with respiratory, cardiovascular, GI, renal, coagulopathy, CNS, skin	IVIG, steroids, enoxaparin	Favorable
12.	Reddy et al., Hyderabad [18]	2021	3	9.5 DOL	CNS:3/3; GI: 3/3; cardiovascular: 1/3 (mild coronary aneurysm);	IVIG, steroids, aspirin	Favorable
13.	Shanker et al., Jaipur [19]	2021	4	22nd DOL	CNS	Steroids, heparin	Favorable
14.	Shaiba et al., Saudi Arabia [10]	2021	2	At birth	Cardiovascular, respiratory, renal	IVIG, steroids, PGE1 transfusion, plasma transfusion	Favorable
15.	Divekar et al., Colorado [20]	2021	1	Not available	Cardiovascular	IVIG	Favorable
16.	Eghbalian et al., Iran [21]	2021	1	9th DOL	GI, respiratory	IVIG+ MP	Favorable
17.	Lima et al., Brazil [22]	2021	1	At birth	Cardiovascular, respiratory, CNS	Pericardiocentesis; mechanical ventilation	Favorable
18.	Bakhle et al., Goa [23]	2021	1	8th DOL	Fever, respiratory	IVIG	Favorable
19.	Schoenmakers et al., Netherland [24]	2021	1	At birth	Birth asphyxia, multiorgan failure, CVS: coronary aneurysms	Steroids IVIG surfactant aspirin	Favorable
20.	Chakravarty et al., Delhi [25]	2022	1	8th DOL	Respiratory, cardiovascular, renal	Steroids, IVIG	Favorable
21.	Gupta et al., India [26]	2022	2	At birth	Respiratory, coagulopathy, cardiac	IVIG, steroids, iNO, bosentan aspirin	One death
22.	Tambekar et al., Maharashtra [8]	2022	3	2nd DOL	Respiratory, cardiovascular, GI, coagulopathy	Steroids, IVIG, LMWH, aspirin	Favorable
23.	Rathore et al., Gujarat [27]	2022	3	All at birth	Respiratory, cardiac, renal, coagulopathy	IVIG, steroids	Favorable
24.	Arun et al., Kerala [28]	2022	1	2nd DOL	CNS, coagulopathy,	IVIG, steroids	Favorable
25.	Voddapelli et al., India [29]	2022	1	At birth	Fever, cardiac, GI, lethargy	IVIG, steroids	Favorable
26.	Costa et al., Italy [30]	2022	1	At birth	Respiratory, CNS, skin	Steroids, IVIG, heparin	Favorable
27.	Malek et al., Dhaka [31]	2022	1	1st DOL	Respiratory, coagulopathy	IVIG	Favorable
28.	Sojisirikul et al., Thailand [32]	2022	1	15th DOL	Respiratory, cardiac, GI	IVIG, steroids	Favorable
29.	Wickramaratne et al., Sri Lanka [33]	2022	4	At birth	Respiratory and renal	Supportive treatment	Nil
30.	This series	June 2021-November 2022	18	1.5 days of life	Respiratory and coagulopathy	IVIG: 8 (44.44%), steroids: 3 (16.67%)	1 (5.55%)

The pathophysiology of MIS-N is postulated to be caused due to exposure to maternal antibodies in utero or transplacental transfer of infection resulting in endogenous production of antibodies or postinfectious immune response to infection in the neonate itself [34]. It is hypothesized that certain antibodies might act against autoantigens and initiate MIS-N in neonates [3]. Hence, it is expected that IVIG or steroids could probably help in the treatment of these neonates. However, it is to be noted that there are no standard recommendations for the use of these drugs in MIS-N.

## Conclusions

MIS-N presents in myriad ways and needs further attention and studies to understand it better. This case series does help us to speculate that coagulopathy can be one of the salient presenting features of MIS-N. With this knowledge, in the immediate post-pandemic era, it is essential that MIS-N be considered in the differential diagnosis of neonates presenting with intracerebral bleeds/infarcts. Since MIS-N is expected to result in immunomodulation with attendant antibodies to self-antigens, the role of human IVIG and steroids needs to be analyzed in more detailed studies.
